# CpG Oligodeoxynucleotides Inhibit RANKL-Induced Osteoclast Formation by Upregulating A20 Deubiquitinase in RAW 264.7 Cells

**DOI:** 10.1155/2022/5255935

**Published:** 2022-08-31

**Authors:** Seong-Kyu Kim, Jung-Yoon Choe, Ki-Yeun Park

**Affiliations:** ^1^Division of Rheumatology, Department of Internal Medicine, Catholic University of Daegu School of Medicine, Daegu, Republic of Korea; ^2^Arthritis and Autoimmunity Research Center, Catholic University of Daegu, Daegu, Republic of Korea

## Abstract

**Objective:**

Activation of toll-like receptor 9 (TLR9) has been proposed to play an inhibitory role in RANKL-induced osteoclastogenesis. A20 deubiquitinase has been found to be related to bone loss. This study investigated the role of CpG oligodeoxynucleotides (CpG-ODNs) through regulation of A20 deubiquitinase in RANKL-induced osteoclast formation.

**Methods:**

RAW 264.7 cells, a murine monocyte-macrophage cell line, were incubated with or without CpG-ODN in the presence of RANKL. Osteoclast-specific genes and their related signaling molecules were measured by real-time quantitative polymerase chain reaction and Western blot assay. Morphological assessment for osteoclast formation was performed using tartrate-resistant acid phosphatase (TRAP) staining and F-actin ring formation staining.

**Results:**

RANKL-induced osteoclast-related genes and proteins, c-Fos, NFATc1, TRAP, cathepsin K, and carbonic anhydrase II were significantly inhibited in RAW 264.7 cells stimulated with CpG-ODN. CpG-ODN attenuated TNF receptor-associated factor 6 (TRAF6), p-I*κ*B*α*, and p-NF-*κ*B expression in RAW 264 cells mediated by RANKL. CpG-ODN increased A20 gene and proteins in time-dependent manner. A20 expression under costimulation with CpG-ODN and RANKL was more decreased than under stimulation with RANKL alone. Cells transfected with A20 siRNA augmented expression of osteoclast-related genes and proteins. Number of TRAP-positive cells transfected with A20 siRNA was higher than those transfected with NC siRNA. A20 expression in cells transfected with IL-1*β* siRNA in the presence of both RANKL and CpG-ODN was more decreased than those with NC siRNA.

**Conclusion:**

This study showed that CpG-ODN suppressed RANKL-induced osteoclast formation through regulation of the A20-TRAF6 axis in RAW 264.7 cells.

## 1. Introduction

Osteoclasts are mature multinucleated cells generated by differentiation and fusion of the monocyte/macrophage precursor lineage [1, 2]. Activation for TNF receptor-associated factor 6 (TRAF6) and downstream signal transduction molecules such as nuclear factor-*κ*B (NF-*κ*B), JNK/AP-1, and extracellular-regulated kinase (ERK) following the binding of receptor activator of nuclear factor-*κ*B ligand (RANKL) to RANK are essential steps in the process of osteoclast differentiation and formation [3, 4]. The activation of nuclear factor of activated T cells c1 (NFATc1) by calcineurin as an important transcription factor to mediate osteoclast differentiation results in numerous osteoclast-specific genes such as tartrate-resistant acid phosphatase (TRAP), carbonic anhydrase II, and cathepsin-K and then promotes bone resorbing multinucleated osteoclast formation.

Activation of toll-like receptors (TLRs) involve the differentiation of osteoclast progenitor cells to multinucleated osteoclasts [5]. Numerous exogenous or endogenous damage-associated molecular patterns (DAMPs) and pathogen-associated molecular patterns (PAMPs) such as Pam3CSK4, lipopolysaccharide (LPS), and flagellin through their ligations with TLR2, TLR4, and TLR5 could mediate the regulation of RANKL-induced osteoclast differentiation and formation. In addition, unmethylated CpG dinucleotides abundant in bacterial DNA bound to TLR9 were found to result in activation of innate immune system [6]. Synthetic CpG oligodeoxynucleotides (CpG-ODN) mimic the immune-stimulatory activity of bacterial DNA [7]. Accumulating evidence has suggested that synthetic CpG-ODN modulates RANKL-induced osteoclast differentiation and formation in mouse bone marrow-derived cells [8–11]. However, the stimulatory or inhibitory osteoclastic effect of CpG-ODN on osteoclast progenitor cells differentiated into osteoclasts has still not been determined.

A20 (known as TNF-*α*-induced protein 3, TNFAIP3), one member of the superfamily of ubiquitin-editing proteases, has emerged as a potent negative regulator through negative feedback loop during NF-*κ*B activation [12, 13]. Several studies demonstrated the inhibitory effect of A20 on inflammatory bone loss through blocking osteoclast differentiation in different conditions. A20 inhibited bone resorption by inducing TRAF6 degradation during formation of LPS-induced multinucleated TRAP-positive cells from human peripheral blood mononuclear cells (PBMCs) [14]. Antiosteoclastic effect of A20 through suppression of TRAF6-dependent autophagy in human periodontal ligament cells (hPDLCs) under hypoxia was also identified [15]. Anti-inflammatory effect of A20 overexpression was found to be associated with blocking bone loss in periodontitis [16]. Despite many studies, the exact mechanism of the negative effect of A20 on the formation of osteoclasts has not yet been established.

There has been a lack of expository studies on regulation of A20 in negatively modulating TLR9-dependent signaling pathways in the process of osteoclastogenesis. Specifically, functional interaction between CpG-ODN and A20 in RANKL-induced osteoclast formation has not been established. Thus, this study investigated whether CpG-ODN, a TLR9 ligand, could control RANKL-induced osteoclast formation through regulation of deubiquitinating protease A20 in RAW 264.7 cells.

## 2. Materials and Methods

### 2.1. Cell Culture

Murine monocyte/macrophage RAW 264.7 cells were purchased from the Korean Cell Line Bank (KCLB, Seoul, Korea). Cells were cultured in *α*-minimal essential medium (*α*-MEM) (Gibco, BRL, Grand Island, USA) supplemented with 10% fetal bovine serum (Hyclone, Logan, USA), 100 U/ml penicillin, and 100 *μ*g/ml streptomycin at 37°C in a 5% humidified CO_2_ incubator. For stimulation, cells were treated with sRANKL (100 ng/ml) for 4 days followed by cotreatment in the presence of CpG-ODN (1 *μ*M) for an additional 2 days. Soluble recombinant mouse RANKL (purity: 98%) and interleukin-1*β* (IL-1*β*) were purchased from PeproTech (Rocky Hill, NJ, USA). Synthetic CpG-ODN 1585 (5′-ggGGTCAACGTTGAgggggg-3′) was purchased from Invitrogen (San Diego, CA, USA).

### 2.2. Quantitative Real-Time Polymerase Chain Reaction (RT-PCR)

Cells (2 × 10^4^/well) were seeded in 24-well plates and pre-incubated with sRANKL (100 ng/ml) for 4 days and then cotreated with CpG-ODN (1 *μ*M) for 2 days. Total RNA was extracted from cells using TRIzol Reagent (Gibco BRL, Grand Island, USA), and complementary DNA (cDNA) was synthesized using a ReverTra Ace-*α*-reverse transcriptase kit (Toyobo, Osaka, Japan). cDNA was subjected to real time PCR using SYBR Green Mix kit (Toyobo, Osaka, Japan) and the CFX Connect real-time PCR system (Bio-Rad Laboratories, Hercules, CA, USA) according to the manufacturers' instructions.

Primers, synthesized at Bionics Company (Seoul, Korea), were as follows: TRAP, forward 5′- AAG GCG AGA GAT TCT TTC CCT G-3′, reverse 5′-ACT GGG GAC AAT TCA CTA GAG C-3′; cathepsin K, forward 5′- CAG CAG AAC GGA GGC ATT GA-3′, reverse 5′-CCT TTG CCG TGG CGT TAT AC-3′; carbonic anhydrase II, forward 5′- CAT TAC TGT CAG CAG CGA GCA-3′, reverse 5′-GAC GCC AGT TGT CCA CCA TC-3′; NFATc1, forward 5′-CTC GAA AGA CAG CAC TGG AGC AT-3′, reverse 5′-CGG CTG CCT TCC GTC TCA TAG-3′; c-Fos, forward 5′-ACC ATG ATG TTC TCG GGT TTC AA-3′, reverse 5′-GCT GGT GGA GAT GGC TGT CAC-3′; A20, forward 5′-TGCCCAGTCTGTAGTCTTCG-3′, reverse 5′-AGTTGTTCAGCCATGGTCCT-3′; and GAPDH, forward 5′-TGC ACC ACC AAC TGC TTA-3′, reverse 5′- GGA TGC AGG GAT GAT GTT C-3′. The relative expression of each gene was analyzed using the 2–*ΔΔ*CT method.

### 2.3. Transfection of siRNA

Cells (1 × 10^4^/well) were seeded in 24-well plates and transfected with mouse A20 siRNA (IDs75258) and mouse IL-1*β* siRNA (IDs68240), mouse TLR9 siRNA (IDs211982), and negative control (NC) siRNA at a final concentration of 100 nM using Lipofectamine RNAiMAX according to the manufacturer's instructions. Briefly, cells were transfected with siRNA using 1 *μ*L of Lipofectamine RNAiMAX dissolved in Opti-MEM media and incubated for 10 min at room temperature. After culture for 48 h, cells were treated with or without sRANKL and CpG-ODN and harvested for subsequent experiments.

### 2.4. Western Blot Analysis

Total proteins were obtained by using radio immunoprecipitation assay (RIPA) buffer (Thermo Scientific, Waltham, MA, USA) containing a protease inhibitor cocktail (protease inhibitor cocktail 1 tablet; Roche Diagnostics, Mannheim, Germany) and centrifuged at 13,000 rpm for 10 min. The protein was measured with BCA Protein Assay Kit by using microplate reader at 562 nm. Equal amounts of protein were separated on 10% SDS-PAGE gel electrophoresis and transferred to nitrocellulose membranes (Bio-Rad Laboratories, Hercules, CA, USA). After blocking with 5% bovine serum albumin, protein was probed with appropriate antibodies.

The primary antibodies used were as follows: anti-TRAP, anti-cathepsin K, anti-c-Fos, anti-NFATc1, anti-TRAF6, and anti-*β*-actin from Santa Cruz Biotechnology (Santa Cruz, CA, USA); anti-carbonic anhydrase II, anti-I*κ*B*α*, and anti-phospho-I*κ*B*α* from Abcam (Cambridge, MA, USA); and anti-IL-1*β* and anti-cleaved IL-1*β* from Cell Signaling Technology (Danvers, MA, USA). The SuperSignal® West Pico chemiluminescent kit (Thermo Scientific, Rockford, IL, USA) was used to detect the target protein. Images were obtained using a ChemiDoc TM XRS system (Bio-Rad Laboratories, Hercules, CA, USA).

### 2.5. Tartrate-Resistant Acid Phosphatase (TRAP) Staining

At differentiation day 8, the cells were stained using a tartrate-resistant acid phosphatase (TRAP) staining kit (Takara Bio, Inc., Shiga, Japan) according to the manufacturer's instructions. Cells were fixed with fixation solution for 5 min at room temperature. After, cells were rinsed twice with distilled water and treated with a substrate solution for acid phosphatase for 45 min at 37°C. TRAP-positive multinucleated cells (MNCs) were visualized by light microscopy (Olympus, Tokyo, Japan).

### 2.6. Actin Ring Staining

To visualize the actin ring, cells (2.5 × 10^4^/well) were seeded on glass coverslips and incubated with sRANKL (100 ng/ml) and CpG-ODN (1 *μ*M) for 8 days. F-Actin rings were stained using Alexa Fluor™ 488 Phalloidin antibody (Thermo Fisher Scientific, Waltham, MA, USA) for 20 min, and the nuclei were stained with DAPI (4′,6-diamidino-2-phenylindole) dye. After the cells were washed twice with PBS containing 0.05% Tween-20, they were mounted in fluorescent mounting medium (Dako, Carpinteria, CA, USA) prior to observation using a fluorescence microscope (TE2000-U, Nikon Instruments Inc., NY, USA).

### 2.7. Statistical Analysis

The data were presented as mean and standard deviation. The statistical differences for target genes including NFATc1, TRAP, cathepsin K, carbonic anhydrase II, c-Fos, A20, and TRAF6 and number of TRAP-positive cells between two groups were calculated by nonparametric Mann–Whitney *U* test. A *p* value < 0.05 was considered statistically significant. The statistical analysis was performed using GraphPad Prism Version 5.04 software GraphPad Software, San Diego, CA, USA.

## 3. Results

### 3.1. CpG-ODN Inhibits RANKL-Induced Osteoclast-Related Factors

**Figure 1:**
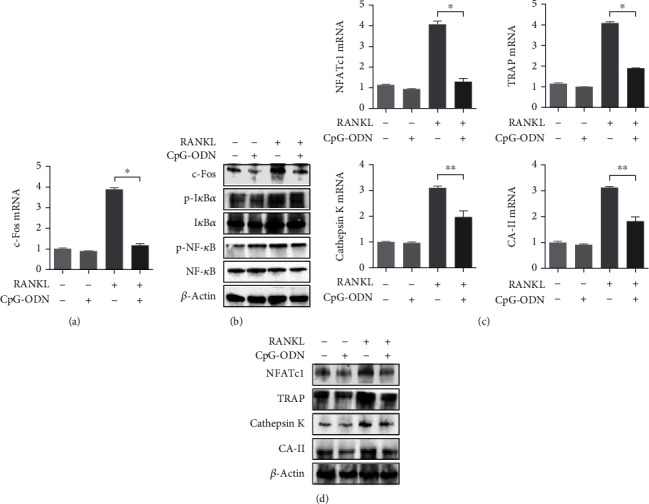
The inhibitory effect of CpG-ODN on osteoclastogenic molecules. (a) c-Fos mRNA expression in RAW 264.7 cells stimulated with either RANKL (100 ng/ml) for 6 days or CpG-ODN (1 *μ*M) for 4 days and both. (b) c-Fos, p-I*κ*B*α*, and p-NF-*κ*B protein expressions in RAW 264.7 cells stimulated with either RANKL or CpG-ODN and both. (c, d) mRNA and protein expression of osteoclast-specific molecules such as NFATc1, TRAP, cathepsin K, and CA-II in RAW 264.7 cells incubated with either RANKL or CpG-ODN and both. Abbreviation: NFATc1: nuclear factor of activated T cells c1; TRAP: tartrate-resistant acid phosphatase; CA-II: carbonic anhydrase-II. Error bars represent standard deviation of the measurement of three separate samples (^∗^*p* < 0.001 and ^∗∗^*p* < 0.05).

We used RAW 264.7 cells to assess the role of CpG-ODN in RANKL-induced osteoclast formation. RAW 264.7 cells were stimulated with RANKL (100 ng/ml) for 6 days in all and with CpG-ODN (1 *μ*M) costimulant for 4 days starting from the second day of RANKL stimulation. First, we examined downstream molecules of the RANK-RANKL signaling pathway including c-Fos, I*κ*B*α*, NF-*κ*B, and osteoclast-specific factors. Experimental cells treated with RANKL alone for 6 days induced c-Fos mRNA expression, which was significantly inhibited in cells cultured with both RANKL and CpG-ODN (Figure 1(a)). However, CpG-ODN alone for 4 days did not affect c-Fos mRNA expression. Consistently, western blotting assay showed that cells incubated with both RANKL and CpG-ODN significantly suppressed c-Fos expression compared with cells incubated with RANKL alone (Figure 1(b)). In addition, phosphorylation of inhibitory binding proteins I*κ*B*α* and NF-*κ*B, which are required for osteoclast formation, was induced by RANKL alone. The same phosphorylation was attenuated in cells stimulated with both RANKL and CpG-ODN (Figure 1(b)).

Stimulation with CpG-ODN for 4 days in RANKL-induced RAW 264.7 cells significantly inhibited mRNA expression of osteoclast-related molecules including NFATc1, TRAP, cathepsin K, and CA-II compared with those treated with RANKL alone for 6 days (Figure 1(c)).  Figure 1(d) shows that protein expression of osteoclast-related markers including NFATc1, TRAP, cathepsin K, and CA-II induced by RANKL were consistently attenuated by additional treatment with CpG-ODN compared with cells treated with RANKL alone.

### 3.2. CpG-ODN in the Presence of RANKL Enhanced Expression of A20 Deubiquitinase

**Figure 2:**
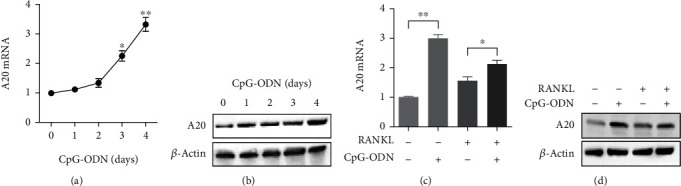
CpG-ODN induced A20 deubiquitinase. (a, b) A20 mRNA and protein expression were measured in RAW 264.7 cells incubated with CpG-ODN (1 *μ*M) for 0 to 4 days. (c, d) A20 mRNA expression in RAW 264.7 cells stimulated with either RANKL (100 ng/ml) or CpG-ODN (1 *μ*M) and both. Error bars represent standard deviation of the measurement of three separate samples (^∗^*p* < 0.05 and ^∗∗^*p* < 0.001).

In addition to the anti-inflammatory action of A20, it is known that A20 deubiquitinase has a crucial role in inhibiting osteoclast differentiation and formation [14–16]. Since CpG-ODN suppressed expression of osteoclast-related genes and proteins under stimulation of RANKL as shown in Figure 1, we examined the A20 expression in RAW 264.7 cells incubated with CpG-ODN. Figures 2(a) and 2(b) show that CpG-ODN induced A20 deubiquitinase mRNA and protein expression in time-dependent manner. A20 deubiquitinase mRNA and protein expression in cells treated with CpG-ODN alone (1 *μ*M) for 4 days were markedly increased compared with non-treated cells (Figures 2(c) and 2(d)). CpG-ODN treatment in RANKL-stimulated RAW 264.7 cells suppressed A20 deubiquitinase mRNA and protein in cells treated with RANKL alone (100 ng/ml) for 6 days.

### 3.3. Deficiency of A20 Deubiquitinase Is Responsible for Osteoclast Formation

**Figure 3:**
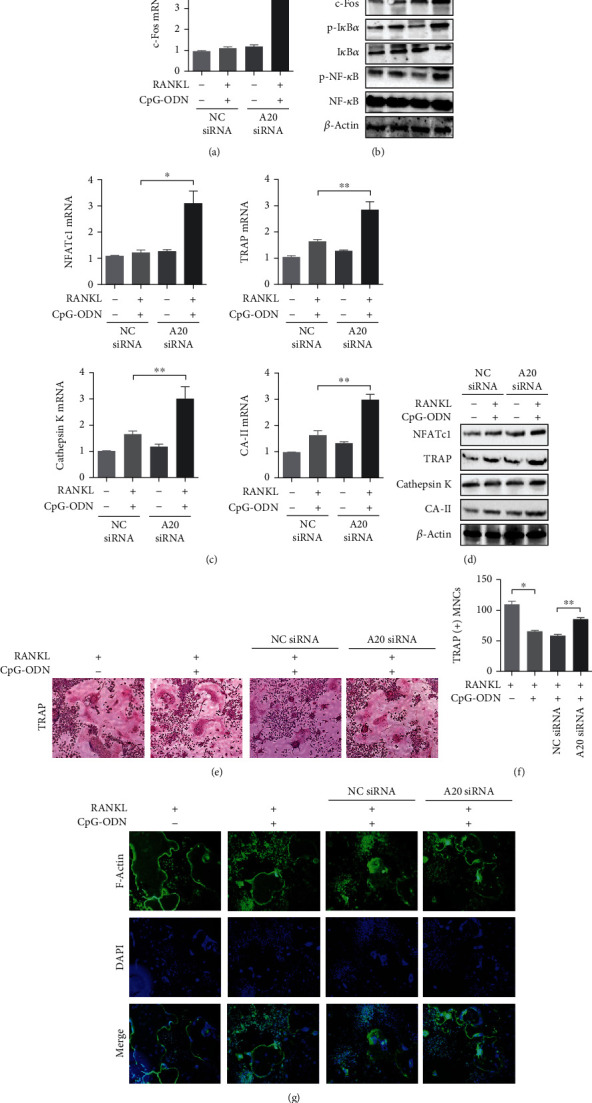
Deficiency of A20 deubiquitinase restored osteoclast formation. (a) c-Fos mRNA expression in A20 knockdown cells under stimulation with both RANKL (100 ng/ml) for 8 days and CpG-ODN for 6 days. (b) c-Fos, p-I*κ*B*α*, and p-NF-*κ*B protein expression in cells transfected with A20 siRNA under stimulation with both RANKL and CpG-ODN. (c, d) Under the same experimental conditions, osteoclast-specific molecules such as NFATc1, TRAP, cathepsin K, and CA-II were measured in A20 siRNA transfected RAW 264.7 cells incubated with both RANKL and CpG-ODN. (e–g) TRAP staining, number of TRAP-positive MNCs, and F-actin ring staining in cells transfected with A20 siRNA in the presence of both RANKL and CpG-ODN were preformed, respectively. Abbreviation: NC: negative control; NFATc1: nuclear factor of activated T cells c1; TRAP: tartrate-resistant acid phosphatase; CA-II: carbonic anhydrase-II. Error bars represent standard deviation of the measurement of three separate samples (^∗^*p* < 0.001 and ^∗∗^*p* < 0.05).

A20 siRNA was used to evaluate the role of A20 deubiquitinase in the osteoclast formation induced by both RANKL and CpG-ODN. In this experiment shown at Figures 3(a) and 3(b), RAW 264.7 cells cultured with both RANKL and CpG-ODN were transfected with either NC siRNA or A20 siRNA. c-Fos mRNA expression was significantly increased in cells transfected with A20 siRNA compared with those transfected with NC siRNA. In addition, cells transfected with A20 siRNA augmented phosphorylation of I*κ*B*α* and NF-*κ*B compared with those transfected with NC siRNA. In the assessment of the effect of A20 on osteoclast-specific markers, silencing A20 in the presence of both RANKL and CpG-ODN increased NFATc1, TRAP, cathepsin K, and CA-II mRNA and protein expression compared with cells transfected with NC siRNA (Figures 3(c) and 3(d)).

TRAP staining assay showed that the size of TRAP-positive cells in RAW 264.7 cells treated with RANKL alone for 8 days was significantly less than the size of those cultured with both RANKL and CpG-ODN (Figure 3(e)). The size of TRAP-positive cells transfected with A20 siRNA was larger than those transfected with NC siRNA. In the comparison of number of TRAP-positive MNCs, CpG-ODN and RANKL cotreatment decreased the number of TRAP-positive MNCs rather than treatment with RANKL alone (Figure 3(f)). The number of osteoclast-like MNCs in A20 silencing cells is much higher than in those transfected without A20 siRNA. F-Actin ring assay found that both RANKL and CpG-ODN produced less fluorescence signal at the peripheral area of osteoclasts treated with than in cells treated with RANKL alone (Figure 3(g)). Cells transfected with A20 siRNA showed more strong fluorescence signal than those transfected with NC siRNA.

### 3.4. IL-1*β* Induced by CpG-ODN in the Presence of RANKL Triggered A20 Deubiquitinase Expression

**Figure 4:**
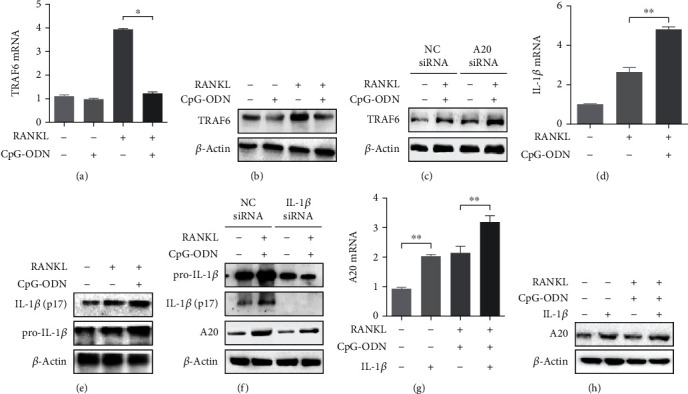
CpG-ODN is involved in the regulation of TRAF6, A20, and IL-1*β*. (a, b) TRAF6 mRNA and protein expression in RAW 264.7 cells under stimulation with either RANKL or CpG-ODN and both. (c) Under the same conditions, TRAF6 protein expression was measured in A20 deficient cells. (d, e) IL-1*β* mRNA and protein expression were augmented by CpG-ODN in the presence of RANKL. (f) IL-1*β* protein expression in cells transfected with A20 siRNA under stimulation with either RANKL or CpG-ODN and both. (g, h) A20 expression was measured in cells under stimulation with both RANKL and CpG-ODN by addition of IL-1*β* (10 ng/ml). Abbreviation: NC: negative control; IL-1*β*: interleukin-1*β*. Error bars represent standard deviation of the measurement of three separate samples (^∗^*p* < 0.001 and ^∗∗^*p* < 0.05).

RANKL-RANK-TRAF6-dependent signaling pathway is essential for osteoclast differentiation and maturation [1, 2]. In the experiment, we evaluated whether CpG-ODN has an effect on TRAF6 in the process of RANKL-induced osteoclast formation. TRAF6 mRNA and protein expression in RAW 264.7 cells treated with both RANKL and CpG-ODN was significantly reduced from those cultured with RANKL alone (Figures 4(a) and 4(b)). TRAF6 expression in RAW 264.7 cells transfected with NC siRNA in the presence of both RANKL and CpG-ODN was increased well beyond the expression of those transfected with A20 siRNA (Figure 4(c)).

A20 is regulated by proinflammatory cytokines such as tumor necrosis factor-*α* (TNF-*α*) and IL-1*β* through negative feedback mechanism [12, 13]. We investigated the role of IL-1*β* in the regulation of CpG-ODN-mediated A20 deubiquitinase. IL-1*β* mRNA and protein expression were increased after stimulation with RANKL (Figures 4(d) and 4(e)). Costimulation with CpG-ODN in the presence of RANKL increased IL-1*β* mRNA and protein expression more than with RANKL alone. Cells transfected with IL-1*β* siRNA in the presence of both RANKL and CpG-ODN showed more reduced expression of A20 protein compared with those transfected with NC siRNA (Figure 4(f)).

We assessed whether recombinant IL-1*β* augmented A20 deubiquitinase expression under stimulation with both CpG-ODN and RANKL (Figures 4(g) and 4(h)). In the preincubated with sRANKL (100 ng/ml) alone or combination with IL-1*β* (10 ng/ml) for 4 day and then cotreated with CpG-ODN (1 *μ*M) for 2 days, recombinant IL-1*β* (10 ng/ml) significantly increased mRNA and protein levels of A20 deubiquitinase in RAW 264.7 cells.

### 3.5. RANKL-Induced Osteoclastogenesis Stimulated by CpG-ODN Was Dependent on TLR9

**Figure 5:**
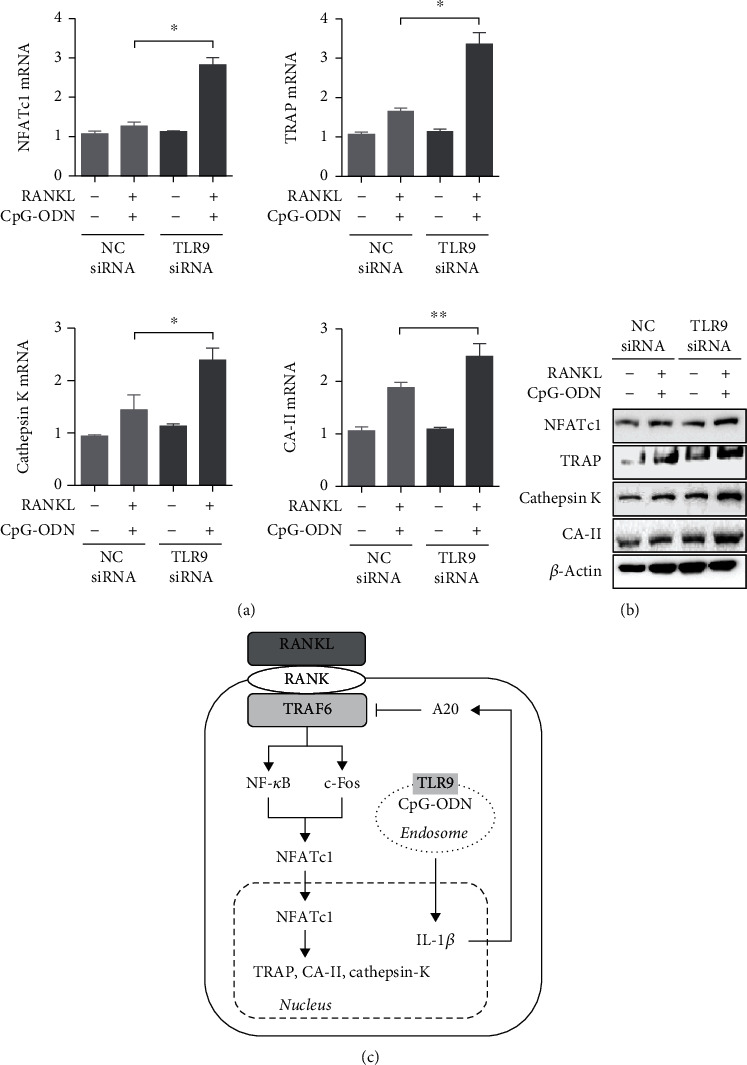
Inhibitory effect of CpG-ODN on RANKL-induced osteoclast formation via TLR9. (a, b) mRNA and protein expressions of osteoclast-specific molecules such as NFATc1, TRAP, cathepsin K, and CA-II were measured in TLR9 siRNA transfected RAW 264.7 cells incubated with both RANKL and CpG-ODN. (c) RANKL-TRAF6-dependent signal pathway initiates differentiation and formation of multinucleated osteoclasts through activation of osteoclast-specific molecules, together with maturation of interleukin-1b. CpG-ODN amplifies IL-1*β* expression, in turn leading to A20 expression. Finally, CpG-ODN-IL-1*β*-A20-TRAF6 signal transduction plays a negative role in RANKL-induced osteoclast formation. Abbreviation: NC: negative control; NFATc1: nuclear factor of activated T cells c1; TRAP: tartrate-resistant acid phosphatase; CA-II: carbonic anhydrase-II. Error bars represent standard deviation of the measurement of three separate samples (^∗^*p* < 0.01 and ^∗∗^*p* < 0.05).

We assessed whether TLR9 was involved in expression of osteoclast-related markers stimulated by both CpG-ODN and RANKL (Figures 5(a) and 5(b)). RAW 264.7 cells transfected with TLR9 siRNA showed higher expression of osteoclast-related markers such as NFATc1, TRAP, cathepsin K, and CA-II than nontransfected cells.

## 4. Discussion

Activation of TLR's response to numerous TLR ligands including exogenous and endogenous PAMPs and DAMPs induces innate immune system. In addition, numerous studies have demonstrated that the binding of TLRs to their TLR ligands in the process of osteoclastogenesis plays a crucial role in either bone resorption or formation in diverse localized and systemic bone diseases [5]. Accumulating evidence has suggested that binding of TLR9 to TLR9 ligand may be responsible for the process of osteoclastogenesis through different mechanisms. TLR stimulation by various microbial products such as LPS, poly(I:C) dsRNA, and CpG-ODN significantly suppressed formation of TRAP-positive MNCs from human osteoclast precursor cells induced by both macrophage colony-stimulating factor (M-CSF) and RANKL, although it promoted NF-*κ*B activation and TNF-*α* production [8]. Amcheslavsky and Bar-Shavit showed that CpG-ODN potently inhibited RANKL-induced osteoclast differentiation from early osteoclast precursors through enhanced production of antiosteoclastogenic factor, IL-12 [10]. Osteoprotegerin (OPG) is a decoy receptor of the receptor activator of NF-B ligand (RANKL), a key regulator of osteoclast differentiation, and blocks RANKL, which stimulates the RANK on the osteoclast surface [1, 2]. Proinflammatory cytokines including TNF-*α* and IL-1 are responsible for bone resorption [17, 18]. Recently, Zheng et al. demonstrated that CpG-ODN showed antiosteoclastic effect through elevation of OPG/RANKL ratio and downregulation of inflammatory cytokines such as TNF-*α*, IL-1*β*, IL-6, and IL-17 in RAW 264.7 cells [19]. CpG-ODN-mediated osteoclastogenesis in the cultures of RANKL-pretreated BMMs was significantly inhibited by neutralizing antibodies for TNF-*α* and the TNF type 1 receptor [20]. Consistent with previous studies, this study also found that CpG-ODN inhibited genes and proteins of osteoclastogenic factors such as NFATc1, TRAP, cathepsin K, and carbonic anhydrase II and suppressed formation of TRAP-positive MNCs from RANKL-induced RAW 264.7 cells. It indicates that TLR9 stimulation through CpG-ODN may be therapeutic or serve as a preventive strategy for osteoclast-related pathologic conditions.

Despite the results for studies presenting the anti-osteoclast effect of CpG-ODN, several other studies demonstrated that CpG-ODN, exerting intracellular activity through TLR9, differentially modulated osteoclastogenesis under different experimental conditions through diverse mechanisms. Proosteoclastic pathways including NF-*κ*B activation, p38/ERK phosphorylation, and production of proinflammatory cytokines such as TNF-*α* and IL-1 in osteoclast precursor cells under stimulation with RANKL and/or CpG-ODN have been proposed as pathological mechanisms for bone resorption [9, 21–23]. Amcheslavsky et al. demonstrated that CpG-ODN showed a dose-dependent suppressive effect on osteoclast precursor cells incubated with RANKL for 4 days, whereas addition of CpG-ODN for the last 30 hours increased production of TRAP-positive MNCs in RANKL-primed BMMs in a dose-dependent manner [9]. CpG-ODN induced ERK phosphorylation required to increase c-Fos expression, which is essential for osteoclast differentiation [11]. Considering the signaling molecules activated by CpG-ODN in generation of osteoclast-like cells, addition of CpG-ODN in RANKL-pretreated cells significantly induced p38 and ERK phosphorylation in TLR9^+/+^ osteoclast progenitor cells and increased production of proinflammatory cytokines such as TNF-*α* and IL-1 and ultimately resulted in osteoclast formation [9]. Consistently, Zou et al. found that the dual effect of CpG-ODN on osteoclastogenesis was observed according to the process of RANKL-induced BMMs in the presence or absence of CpG-ODN [20]. Stimulation with both CpG-ODN and RANKL suppressed osteoclast formation by downregulation of M-CSF receptor in the early phase of osteoclastogenesis, whereas osteoclastogenesis was increased by induction of CpG-ODN-mediated TNF-*α*. Another study demonstrated that CpG-ODN did not have any potency to regulate osteoclast formation. LPS for TLR4, but not CpG-ODN for TLR9, significantly induced generation of TRAP-positive MNCs from bone marrow cells in Src homology 2-domain phosphatase-1-defective mice in the presence of M-CSF [24].

It has been well established that the deubiquitinating protease A20 inhibited osteoclast differentiation and formation from diverse osteoclast precursor cells caused by various stimuli including LPS, nicotine, or hypoxia [14–16]. The antiosteoclastogenic effect of A20 on bone resorption is tightly regulated by different action mechanisms in human inflammatory and autoimmune diseases. Yan et al. investigated whether A20 displayed an antiosteoclastogenic potency in bone resorption in periodontitis [15]. As the oxygen concentration decreased, A20 was dose-dependently attenuated in hPDLCs. A20 suppressed osteoclast formation through reduced polyubiquitination at K63 and enhanced polyubiquitination at K48 in TRAF6 under hypoxia. It implicated that downregulation of TRAF6-dependent autophagy by A20 was responsible for inhibition of osteoclastogenesis in BMMs cultured in the conditioned medium from hPDLCs. Similarly, A20 overexpression using recombinant adenovirus encoding A20 suppressed the number of TRAP-positive osteoclasts differentiated from BMMs using conditioned medium from nicotine- and LPS-induced hPDLCs through inhibition of NF-*κ*B activation and p38 phosphorylation [16]. As part of one crucial step in osteoclastogenesis, TRAF6 acts as a crucial adaptor protein that mediates the downstream signal transduction pathways of RANKL-RANK binding through recruitment to the intracytoplasmic domain of RANK, leading to activation of NF-*κ*B, MAPK family including JNK, ERK, and p38/AP-1, and Src/AKT [1–3]. Mabilleau et al. found that A20 hampered capacity for bone resorption in LPS-induced osteoclasts in late stage of osteoclastogenesis through TRAF6 degradation and NF-*κ*B inhibition, although addition of LPS significantly induced TRAP-positive MNCs in the presence of RANKL and M-CSF [14]. In the present study, we also found that CpG-ODN significantly induced A20 expression in RANKL-induced osteoclast formation from RAW 264.7 cells through blockage of TRAF6. Inhibition of A20 using A20 siRNA induced osteoclast-related genes and increased the number of TRAP-positive cells in the stimulation with RANKL and CpG-ODN through increased TRAF6 expression. Upregulation of the deubiquitinating protease A20 by CpG-ODN might be implicated in the inhibitory effect on osteoclastogenesis.

Bacterial DNA serves as a PAMP that is responsible for activation of immune system and has been shown to induce the innate immune response through TLR9, in turn, resulting in resistance against pathogens and sequential initiation of the adaptive immune response. CpG-ODN has been demonstrated to exert immune stimulatory effect and secretion of inflammatory cytokines [7]. Diverse inflammatory cytokines including TNF-*α*, IL-6, IL-1*β*, and transforming growth factor-*β* were considered key mediators for bone resorption or formation [4, 25]. Bacterial nucleic acid including CpG-ODN has been found to induce an inflammatory response through release of proinflammatory cytokines including TNF-*α*, IL-1*β*, IL-6, IL-12p70, and IL-23, then leading to initiation of chronic inflammatory diseases [9, 26, 27]. Cells from TLR9-deficient mice showed no release of proinflammatory cytokines including TNF-*α*, IL-6, and IL-12p40 to CpG-DNA [6]. Although IL-1 alone cannot induce osteoclast differentiation from osteoclast precursor BMMs, IL-1 showed a potency to involve in RANKL-induced osteoclast formation [28]. In the present study, we found that expression of IL-1*β* mRNA and protein was significantly increased in RANKL-stimulated RAW 254.7 cells in the presence of CpG-ODN compared with those stimulated with RANKL alone. Enhanced IL-1*β* expression by CpG-ODN stimulation significantly induced the expression of A20 that plays as an inhibitor of NF-*κ*B activation and eventually inhibited osteoclast formation through blockage of TRAF6. Similarly, prolonged treatment with LPS to PBMCs induced A20 expression and TRAF6 degradation, then leading to attenuated bone resorption at late stage osteoclastogenesis, although LPS-induced TNF-*α* expression stimulated osteoclast formation at the early phase [14]. However, three CpG-ODNs inhibited secretion of proinflammatory cytokines including TNF-*α*, IL-1*β*, IL-6, and IL-17 in RAW 264.7 cells at early (6 h) or late (24 h) stages [19]. Although the expression of proinflammatory cytokines by CpG-ODN was increased, CpG-ODN still inhibited the differentiation and proliferation of osteoclasts. The inflammatory cytokines were variously expressed by different conditions such as the timing and duration of stimulation of CpG-ODN. This difference in the expression of inflammatory cytokines may suggest the possibility that osteoclast differentiation may be affected.

There are largely two distinct classes of CpG-ODNs. Type A CpG-ODNs including ODN 1585 are characterized by induction of high interferon-*α* (IFN-*α*) production but not weak stimulation of NF-*κ*B signaling by immature plasmacytoid dendritic cells [29, 30]. In contrast, type B CpG-ODNs including ODN 1826 strongly stimulate B cell activation and NF-*κ*B singling but weakly induce IFN-a secretion [29, 30]. It has well recognized that type I IFNs including IFN-*α*, IFN-*β*, and IFN-*γ* downregulate osteoclast differentiation and activation, whereas NF-*κ*B signaling plays a crucial role in the regulation of bone loss [18]. Chang et al. found that murine specific CpG-ODN 1826 inhibited osteoclast formation but not differentiation [31]. Simultaneous or additional administration of CpG-OND 1826 under RANKL stimulation showed a dual effect on osteoclastogenesis [9]. Other study demonstrated that ODN 1826 inhibited RANKL-induced osteoclast differentiation by increased degradation of c-Fos [11]. Depending on the class of CpG-ODNs and experimental conditions of their uses, it can be assumed that they can have differential effects on the osteoclast differentiation or formation. There is not enough evidence on how CpG-ODN 1585 affects osteoclast formation. Our hypothesis is that type A CpG-ODN may inhibit osteoclastogenesis as it enhances the production of IFN-*α* showing inhibitory function of osteoclast formation. Therefore, we used type A CpG-ODN 1585 in this study and confirmed that CpG-ODN 1585 also inhibited osteoclast differentiation and formation through enhancement of A20.

In conclusion, CpG-ODN inhibited differentiation and formation of osteoclast precursor cells in RAW 264.7 cells cultured with RANKL into osteoclasts (Figure 5(c)). Although there have been some results suggesting that CpG-ODN could increase bone resorption in osteoclast precursors [9, 20], the antiosteoclastogenic effect of CpG-ODN confirmed in the present study is consistent with previous studies [8, 10, 11, 19]. Osteoclast progenitor cells that differentiate into mature osteoclasts are affected by numerous pro- and antiosteoclastic cytokines and molecules such as TNF-*α*, IL-6, IFN-*γ*, or IL-4, which regulate osteoclast function [4, 25]. In addition, the novel finding of this study is that the antiosteoclastogenic effect of CpG-ODN is derived by upregulation of A20 deubiquitinase induced by increased expression of IL-1*β*. In turn, inhibition of TRAF-6 by enhancement of A20 is responsible for the antiosteoclastic effect of CpG-ODN in RANKL-induced osteoclast formation. Further elucidation of novel pathways for the proosteoclastic and antiosteoclastic effects of CpG-ODN is needed.

## Data Availability

The data underlying this article will be shared on reasonable re-quest to the corresponding author.

## References

[1] Boyle W. J., Simonet W. S., Lacey D. L. (2003). Osteoclast differentiation and activation. *Nature*.

[2] Ono T., Nakashima T. (2018). Recent advances in osteoclast biology. *Histochemistry and Cell Biology*.

[3] Boyce B. F., Xiu Y., Li J., Xing L., Yao Z. (2015). NF-*κ*B-mediated regulation of osteoclastogenesis. *Endocrinology and Metabolism*.

[4] Cappariello A., Maurizi A., Veeriah V., Teti A. (2014). *The Great Beauty* of the osteoclast. *Archives of Biochemistry and Biophysics*.

[5] Souza P. P. C., Lerner U. H. (2019). Finding a toll on the route: the fate of osteoclast progenitors after toll-like receptor activation. *Frontiers in Immunology*.

[6] Hemmi H., Takeuchi O., Kawai T. (2000). A Toll-like receptor recognizes bacterial DNA. *Nature*.

[7] Krieg A. M. (2002). CpG motifs in bacterial DNA and their immune effects. *Annual Review of Immunology*.

[8] Takami M., Kim N., Rho J., Choi Y. (2002). Stimulation by toll-like receptors inhibits osteoclast differentiation. *Journal of Immunology*.

[9] Amcheslavsky A., Hemmi H., Akira S., Bar-Shavit Z. (2005). Differential contribution of osteoclast- and osteoblast-lineage cells to CpG-oligodeoxynucleotide (CpG-ODN) modulation of osteoclastogenesis. *Journal of Bone and Mineral Research*.

[10] Amcheslavsky A., Bar-Shavit Z. (2006). Interleukin (IL)-12 mediates the anti-osteoclastogenic activity of CpG- oligodeoxynucleotides. *Journal of Cellular Physiology*.

[11] Amcheslavsky A., Bar-Shavit Z. (2007). Toll-like receptor 9 ligand blocks osteoclast differentiation through induction of phosphatase. *Journal of Bone and Mineral Research*.

[12] Shembade N., Ma A., Harhaj E. W. (2010). Inhibition of NF-*κ*B signaling by A20 through disruption of ubiquitin enzyme complexes. *Science*.

[13] Boone D. L., Turer E. E., Lee E. G. (2004). The ubiquitin-modifying enzyme A20 is required for termination of Toll-like receptor responses. *Nature Immunology*.

[14] Mabilleau G., Chappard D., Sabokbar A. (2011). Role of the A20-TRAF6 axis in lipopolysaccharide-mediated osteoclastogenesis. *The Journal of Biological Chemistry*.

[15] Yan K., Wu C., Ye Y. (2020). A20 inhibits osteoclastogenesis via TRAF6-dependent autophagy in human periodontal ligament cells under hypoxia. *Cell Proliferation*.

[16] Hong J. Y., Bae W. J., Yi J. K., Kim G. T., Kim E. C. (2016). Anti-inflammatory and anti-osteoclastogenic effects of zinc finger protein A20 overexpression in human periodontal ligament cells. *Journal of Periodontal Research*.

[17] Walsh M. C., Kim N., Kadono Y. (2006). Osteoimmunology: interplay between the immune system and bone metabolism. *Annual Review of Immunology*.

[18] Amarasekara D. S., Yun H., Kim S., Lee N., Kim H., Rho J. (2018). Regulation of osteoclast differentiation by cytokine networks. *Immune Network*.

[19] Zheng Y., Yu W., Li H. (2020). CpG oligodeoxynucleotides inhibit the proliferation and osteoclastic differentiation of RAW264.7 cells. *RSC Advances*.

[20] Zou W., Schwartz H., Endres S., Hartmann G., Bar-Shavit Z. (2002). CpG oligonucleotides: novel regulators of osteoclast differentiation. *The FASEB Journal*.

[21] Zou W., Amcheslavsky A., Bar-Shavit Z. (2003). CpG oligodeoxynucleotides modulate the osteoclastogenic activity of osteoblasts via Toll-like receptor 9. *The Journal of Biological Chemistry*.

[22] Matsumoto M., Sudo T., Saito T., Osada H., Tsujimoto M. (2000). Involvement of p38 mitogen-activated protein kinase signaling pathway in osteoclastogenesis mediated by receptor activator of NF-*κ*B ligand (RANKL). *The Journal of Biological Chemistry*.

[23] Lee S. E., Woo K. M., Kim S. Y. (2002). The phosphatidylinositol 3-kinase, p38, and extracellular signal-regulated kinase pathways are involved in osteoclast differentiation. *Bone*.

[24] Hayashi S., Tsuneto M., Yamada T. (2004). Lipopolysaccharide-induced osteoclastogenesis in Src homology 2-domain phosphatase-1-deficient viable motheaten mice. *Endocrinology*.

[25] Zupan J., Jeras M., Marc J. (2013). Osteoimmunology and the influence of pro-inflammatory cytokines on osteoclasts. *Biochemia Medica*.

[26] Fuss I. J., Becker C., Yang Z. (2006). Both IL-12p70 and IL-23 are synthesized during active Crohn’s disease and are down-regulated by treatment with anti-IL-12 p40 monoclonal antibody. *Inflammatory Bowel Diseases*.

[27] Nigar S., Yamamoto Y., Okajima T. (2017). Synergistic oligodeoxynucleotide strongly promotes CpG-induced interleukin-6 production. *BMC Immunology*.

[28] Kim J. H., Jin H. M., Kim K. (2009). The mechanism of osteoclast differentiation induced by IL-1. *Journal of Immunology*.

[29] Krug A., Rothenfusser S., Hornung V. (2001). Identification of CpG oligonucleotide sequences with high induction of IFN-*α*/*β* in plasmacytoid dendritic cells. *European Journal of Immunology*.

[30] Verthelyi D., Ishii K. J., Gursel M., Takeshita F., Klinman D. M. (2001). Human peripheral blood cells differentially recognize and respond to two distinct CPG motifs. *Journal of Immunology*.

[31] Chang J. H., Chang E. J., Kim H. H., Kim S. K. (2009). Enhanced inhibitory effects of a novel CpG motif on osteoclast differentiation via TREM-2 down-regulation. *Biochemical and Biophysical Research Communications*.

